# 15-Year Experience of Distal Pancreatectomy with Celiac Axis Resection (DP-CAR) for Pancreatic Cancer—A Korean Nationwide Investigation

**DOI:** 10.3390/cancers15153850

**Published:** 2023-07-28

**Authors:** So Jeong Yoon, Sang-Jae Park, Yoo-Seok Yoon, Tae-Ho Hong, Jin-Young Jang, Hee Joon Kim, Jin Seok Heo, Dae Wook Hwang, In Woong Han

**Affiliations:** 1 Division of Hepatobiliary-Pancreatic Surgery, Department of Surgery, Samsung Medical Center, Sungkyunkwan University School of Medicine, Seoul 06351, Republic of Korea; sojeong.yoon@samsung.com (S.J.Y.); jsheo.md@gmail.com (J.S.H.); 2 Center for Liver Cancer, National Cancer Center, Ilsan 10408, Republic of Korea; spark@ncc.re.kr; 3 Department of Surgery, Seoul National University Bundang Hospital, Seoul National University College of Medicine, Bundang 13620, Republic of Korea; arsyun@gmail.com; 4 Department of HBP Surgery, Department of Surgery, Seoul St. Mary’s Hospital, College of Medicine, The Catholic University, Seoul 06591, Republic of Korea; gshth@catholic.ac.kr; 5 Department of Surgery, Seoul National University Hospital, Seoul National University College of Medicine, Seoul 03080, Republic of Korea; jangjy4@gmail.com; 6 Division of Hepato-Pancreato-Biliary Surgery, Department of Surgery, Chonnam National University Hospital, Gwangju 61469, Republic of Korea; heejoonkim@chonnam.ac.kr; 7 Division of Hepatobiliary and Pancreatic Surgery, Department of Surgery, Asan Medical Center, University of Ulsan College of Medicine, Seoul 05505, Republic of Korea

**Keywords:** pancreatic cancer, pancreatectomy, celiac axis resection, prognosis, neoadjuvant chemotherapy

## Abstract

**Simple Summary:**

Distal pancreatectomy with celiac axis resection (DP-CAR) is a procedure for achieving curative resection in pancreatic body or tail cancer involving celiac axis. The present study aimed to investigate surgical and oncologic outcomes of DP-CAR using a Korean nationwide database. A total of 75 patients who underwent DP-CAR between 2007 and 2021 were included in the study. The major complication rate was 26.7%, with two (2.7%) procedure-related mortalities. There were 10 (13.3%) patients with gastropathy and two (2.7%) patients with hepatic ischemia. The median recurrence-free survival was seven months and the median overall survival was 19 months. In neoadjuvant treatment group (*n* = 42), the maximal preoperative value of standardized uptake from positron emission tomography was an independent factor for recurrence-free survival. The decrease in carbohydrate antigen 19-9 level predicted prolonged overall survival in patients with neoadjuvant treatment. We identified that DP-CAR could be a potential option for borderline resectable or locally ad-vanced pancreatic cancer in selected patients.

**Abstract:**

Background: As systemic treatment for pancreatic cancer advances, distal pancreatectomy with celiac axis resection (DP-CAR) has been considered a curative-intent surgical option for advanced pancreatic cancer. This study aimed to review the surgical and oncologic outcomes of patients undergoing DP-CAR based on Korean nationwide data. Methods: We collected the data of patients who underwent DP-CAR for pancreatic cancer between 2007 and 2021 at seven major hospitals in Korea. The clinicopathological characteristics, postoperative complications, and data on the survival of the patients were retrospectively reviewed. Logistic regression analysis was performed to identify risk factors for postoperative complications and survival. Results: A total of 75 patients, consisting mainly of borderline resectable (*n* = 32) or locally advanced (*n* = 30) pancreatic cancer, were included in the analysis. Forty-two (56.0%) patients underwent neoadjuvant treatment (NAT). Twenty (26.7%) patients experienced Clavien–Dindo grade ≥ 3 complications, including four patients with ischemic gastropathy, two with hepatic ischemia, and two procedure-related mortalities. Neoadjuvant chemotherapy increased the risk of postoperative complications (*p* = 0.028). The median recurrence-free and overall survival were 7 and 19 months, with a 5-year survival rate of 13% and 24%, respectively. In the NAT group, a decrease in CA 19-9 and the post-NAT maximum standardized uptake value (SUVmax) in positron emission tomography were associated with survival after surgical resection. Conclusions: Despite the possibility of major complications, DP-CAR could be a feasible option for achieving curative resection with fair survival outcomes in patients with borderline resectable or locally advanced pancreatic cancer. Further studies investigating the safety of the procedure and identifying proper surgical candidates with potential survival gains are necessary.

## 1. Introduction

Pancreatic cancer is a highly aggressive malignancy and only 15~20% of patients are found to have resectable or borderline resectable pancreatic cancer (BRPC) [1]. Some of the remaining patients present with locally advanced pancreatic cancer (LAPC), which is basically a nonmetastasized disease with extensive involvement of vascular structures, resulting in the possibility of non-curative resection [2]. Recently, neoadjuvant treatment (NAT) has become an option for BRPC or LAPC with an expectation of tumor downstaging and proceeding to curative-intent surgery.

Distal pancreatectomy with celiac axis resection (DP-CAR) is a procedure for achieving R0 resection following chemotherapy, particularly for potentially resectable pancreatic body cancer. The procedure has the concept of maintaining hepatic and gastric flow by backflow through the inferior pancreaticoduodenal artery and pancreaticoduodenal arcade [3]. Hepatic and gastric ischemia are procedure-specific complications that could be associated with mortality, but there are limited data on effective methods to prevent complications [4,5].

There have been reports from several research groups around the world. A Japanese group analyzed the outcomes of 72 patients with DP-CAR, and the median survival was 17.5 months with a 4.2% in-hospital death rate [6]. In 2019, a European multicenter study including 71 patients reported a median survival of 20 months with a 90-day mortality rate of 16% [7]. Overall, the authors suggested that the procedure achieved fair survival outcomes and acceptable complication rates. In the present study, we aimed to review the outcomes of DP-CAR using Korean nationwide data and identify proper candidates for the procedure.

## 2. Materials and Methods

### 2.1. Data Collection

A total of 75 patients underwent DP-CAR for pancreatic cancer between January 2007 and December 2021 at seven major hospitals in Republic of Korea. Demographic, clinicopathological data, and medical records during the follow-up periods were retrospectively reviewed. The preoperative data included laboratory results such as carbohydrate antigen 19-9 (CA 19-9) and the receipt of neoadjuvant chemotherapy or chemo-radiotherapy. The recent National Comprehensive Cancer Network guidelines [8] were used to assess resectability of tumor.

In patients who underwent NAT, the NAT response was assessed using three modalities: serum CA 19-9 levels, computed tomography (CT) scans, and positron emission tomography (PET)-CT after NAT. A biologic response was defined as any decrease in CA 19-9 after NAT compared to the initial value. A radiologic response was determined when there was a change in resectability status according to NCCN guidelines. The metabolic response was measured by the maximal standardized value of fluorodeoxyglucose (FDG) uptake, which is also called SUVmax. For patients in whom arterial resection was planned preoperatively, CT angiography was performed to evaluate both tumor status and vascular anatomy.

Data on combined organ resection or the resection and reconstruction of major vessels were collected from surgical records. Before the resection of celiac trunk, the operator first clamped the common hepatic artery (CHA) at gastroduodenal artery (GDA) bifurcation in order to check the backflow from GDA to the proper hepatic artery (PHA). If the backflow was not clearly detected by palpation, intraoperative ultrasonography was used to identify blood flow to the liver if available. In cases with inadequate hepatic flow or ischemic change of liver parenchyme, arterial reconstruction from aorta to PHA was performed. A thorough inspection and palpation of the reconstructed vessel was made by the operator before the closure of the abdomen. In cases with vascular reconstruction, intraoperative intravenous heparin was injected during vessel anastomosis.

Postoperative complications were graded based on the Clavien-Dindo classification. Procedure-related gastropathy included delayed gastric emptying (DGE), gastric ischemia, or perforation identified on postoperative CT scans. Hepatic ischemia was diagnosed when there was a sign of ischemia on the CT scans with elevated aminotransferases in liver function tests. The definition and grading of postoperative pancreatic fistula (POPF) were based on the International Study Group of Pancreatic Surgery (ISGPS) criteria [9]. Tumor size, lymph node status, tumor differentiation, and resection margin status were reported in the postoperative pathology reports.

The dates of cancer recurrence and death were reviewed to evaluate oncologic outcomes. Recurrence was determined when a patient presented with an elevated CA 19-9 level with suspicious lesions on CT scans during postoperative surveillance. Recurrence-free survival (RFS) and overall survival (OS) were measured by the time from surgery to the date of the event (recurrence or death).

### 2.2. Statistical Analysis

The demographic and clinicopathological data are described as the mean ± standard deviation or frequency with percentile for categorical variables. Binary logistic regression was performed to identify risk factors for postoperative complications. In risk factor analysis, an odds ratio (OR) was presented with the 95% confidence interval (CI). A Kaplan–Meier survival graph was drawn to investigate survival of the patient cohort. Cox regression analysis was performed to investigate factors related to survival, and the hazard ratio (HR) with 95% CI was calculated for each variable. Variables with a *p*-value of less than 0.1 in the univariable analysis were included in the multivariable analysis, and *p*-values of less than 0.05 were considered statistically significant. All statistical analyses were conducted using IBM SPSS software (version 25, SPSS Inc., Chicago, IL, USA).

## 3. Results

The preoperative clinical data of the patients are summarized in Table 1. The mean age at operation was 62.6 years, and 50.6% of the patients were male. Thirty-one (41.3%) patients had elevated CA 19-9 levels, and 42 (56.0%) patients received NAT. Regarding resectability, most of the patients had BRPC (*n* = 42, 56.0%) or LAPC (*n* = 30, 40.0%). Some of the patients in the NAT group who had LAPC showed radiologic responses, and the proportion of patients with BRPC increased after NAT from 42.9% to 61.9%.

Table 2 shows the operative findings and final pathology of the patients. Two (2.7%) patients underwent common hepatic artery (CHA) reconstruction using a cadaveric graft. The mean operation time was 298.4 min, and 18 (24.0%) patients needed red blood cell transfusions. According to final pathology reports, no residual tumor was identified in two (2.7%) patients, and five (6.7%) patients presented with M1 disease: one with liver metastasis, three with peritoneal seeding nodules, and one with a positive para-aortic LN. R0 resection was achieved in 49 (65.3%) patients, and the rate of R0 resection was higher in patients with NAT than those with upfront surgery (73.8% vs. 54.5%, *p* = 0.082).

In terms of surgical outcomes, the overall complication rate was 62.7% (Table 3). Major complications, in which the Clavien–Dindo grade was III or higher, occurred in 26.7% of the patients. There were two (2.7%) patients with re-operation. The first case underwent primary repair of stomach and insertion of feeding jejunostomy due to gastric perforation at postoperative day 42. The second case underwent bleeding control for hemorrhage from drain catheter removal site at postoperative day 3. Ten (13.3%) patients had gastropathy, including six with delayed gastric emptying, three with gastric ischemia, which resolved with non-operative management, and one with gastric perforation, which was treated with re-operation. Two (2.7%) patients presented with hepatic ischemia, which required only conservative management. In these patients, the vital signs and laboratory markers, including blood count and liver function test, were closely monitored in the intensive care unit. Oral diet was encouraged if tolerable, and intravenous hydration was conducted in order to avoid dehydration. In all cases, including those with CHA reconstruction, there was no thrombotic occlusion of arteries. Four (5.3%) patients had portal vein (PV) thrombosis, which required PV stent insertion by angiographic intervention. There were two (2.7%) procedure-related mortalities. The first patient had a clinically relevant postoperative pancreatic fistula (CR-POPF) and gastric ischemia and died from sepsis involving multiple organ failure. The cause of death in the second mortality case was septic shock related to CR-POPF. Neoadjuvant chemotherapy was identified as an independent factor in risk factor analysis for major complications (Table 4) (OR: 4.445, 95% CI: 1.176–16.795, *p* = 0.028).

**Figure 1:**
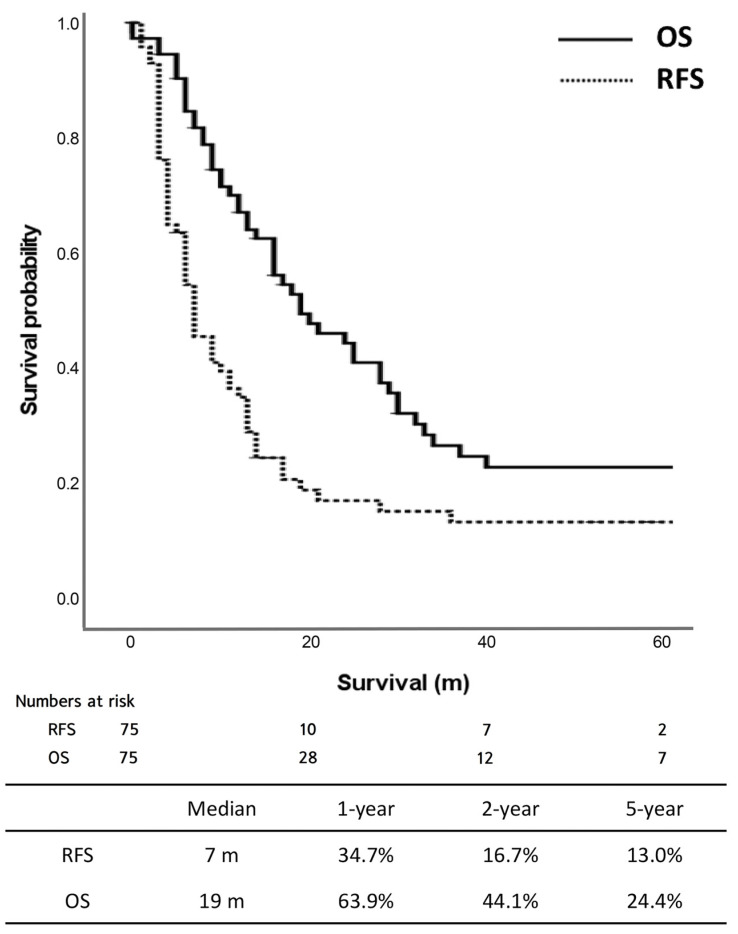
Recurrence-free survival and overall survival in all patients (*n* = 75).

Regarding oncologic outcomes, Figure 1 shows the RFS and OS of the cohort. The median RFS was seven months, with the 5-year RFS rate of 13.0%. The median OS was 19 months, and the 5-year OS rate was 24.4%.

Risk factor analysis was performed to identify preoperative parameters associated with survival after NAT followed by DP-CAR (Table 5). For RFS, the preoperative SUVmax on PET-CT was an independent risk factor in multivariable analysis (HR: 1.548, 95% CI: 1.061–2.260, *p* = 0.024). In the analysis of OS, a decrease in CA 19-9 after NAT, also referred to as a biologic response, was a significant protective factor (HR: 0.114, 95% CI: 0.017–0.772, *p* = 0.026).

## 4. Discussion

The origin of DP-CAR can be traced to the Appleby procedure, which was performed as radical surgery for gastric cancer [10]. As the procedure was applied to pancreatic cancer, surgeons tried to preserve the stomach based on the existence of collateral blood flow [11]. A series of previous studies reported the feasibility of DP-CAR, particularly in patients with neoadjuvant and adjuvant chemotherapy for BRPC or LAPC [6,7,12,13,14,15,16,17,18]. However, the procedure could be accompanied by specific and sometimes fatal complications, as well as hepatic and gastric ischemia, and only limited data are available on the prevention and management of these adverse events. To assess the oncologic efficacy and surgical safety of DP-CAR, we aimed to review the overall outcomes of this procedure using Korean nationwide data.

Several previous studies reported the outcomes of DP-CAR and explored the clinical implications of the procedure (Table S1). The complication rate of about 40% in the previous studies is higher than that of other major abdominal surgeries. Although the outcomes between the study groups are not directly comparable because of cohort heterogeneity, our data showed relatively low complication and mortality rates, with comparable survival outcomes. Particularly compared to studies from Japanese [6] and European [7] groups, in which the number of included patients and the proportion of NAT were similar to those of the present study, the incidence of major complications, including procedure-related ischemic complications, was lower in our cohort even without preoperative radiologic preparations. Gastric perforation occurs due to hemodynamic changes after resection of the celiac axis and left gastric artery. The reported incidence ranges from 3.7% to 29% [7,12,13,19]. In our study, among four (5.3%) patients with ischemic gastropathy, one (1.3%) patient had perforation requiring surgical exploration. In a previous study reporting a DP-CAR case series [20], the authors performed preoperative angiographic embolization of the celiac trunk for some patients, and gastric ischemia did not occur among them. Among two patients who did not undergo preoperative angiography, one patient experienced gastric perforation. They argued that preconditioning of the celiac axis by angiographic occlusion would prevent postoperative ischemic complications. A recent study from Russia reported hemodynamic changes after DP-CAR using perioperative CT scans and intraoperative ultrasound [19], but there was no statistical feature regarding postoperative changes in gastric flow, particularly in the right gastroepiploic artery. Considering that there has been no preceding research identifying preventive strategies for gastric perforation, further research is needed to explore potential solutions for ischemic gastropathy following DP-CAR.

The incidence of hepatic ischemia varied from 0% to 25% in previous studies [7,17,19,21]. Even in reports with relatively higher rates of hepatic ischemia, severe hepatic infarction or abscesses requiring invasive procedures, such as percutaneous drainage, accounted for only a minor proportion of the patients. Since most previous studies were performed using a retrospective design, and the application of preoperative angiography or arterial reconstruction during surgery varied among studies, the risk and protective factors for hepatic ischemia remain unclear. Several reports explored the efficacy of preoperative hepatic artery embolization in preventing DP-CAR-related hepatic infarction. The results somewhat differed between the studies, with some authors suggesting preoperative embolization as a feasible option for patients undergoing DP-CAR [4,22] and others arguing that the procedure had no impact on ischemic complications [7,21]. Despite the inconsistency, the common implication would be that preservation of hepatic flow from pancreaticoduodenal arcade is a basal condition for preventing severe hepatic ischemia. Therefore, the meticulous assessment of hepatic flow during surgery using palpation or intraoperative ultrasound should be considered for patients with CHA resection.

In terms of the oncologic benefit of DP-CAR, selecting the proper candidates who might derive benefit from this highly invasive procedure is most crucial. Particularly in the era of NAT for pancreatic cancer, the issue of determining appropriate surgical indications is important yet inconclusive. We evaluated the feasibility of three indicators for predicting oncologic outcomes in patients with NAT, biologic (decrease in CA 19-9 levels), radiologic (tumor geography), and metabolic (change in FDG uptake) responses. In the analysis, a biologic response was an independent factor predicting favorable OS in patients with NAT, followed by DP-CAR. Consecutive measurements of CA 19-9 levels have been utilized as a predictor of operability and survival in patients with NAT [23]. According to some previous studies including patients with initially unresectable pancreatic cancer, CA 19-9 response was related to successful R0 resection and prolonged OS after chemotherapy followed by surgical resection [24,25]. Since the proper cut-off value or the degree of CA 19-9 decrease has been inconsistent between studies, further research with a sufficient number of patients with serial measurements of CA 19-9 should be performed in order to concretize the utilization of the marker as a predictor for successful oncologic resection.

A radiologic response can be assessed by the response evaluation criteria for solid tumors (RECIST) guidelines [26] or changes in NCCN resectability criteria. It was reported that RECIST did not reflect the response of BRPC to NAT and poorly predicted survival outcomes [27,28]. We reviewed the change in resectability status rather than RECIST, but it was not associated with either RFS or OS. These results suggest that radiologic response alone could not be a critical clue for determining the timing of curative resection. To overcome the limitations of the radiologic evaluation of BRPC or LAPC in terms of NAT response, metabolic activity, which is defined by FDG uptake on PET-CT, has been a topic of research. In the present study, the immediate preoperative SUVmax, rather than changes in the value, was a predictive factor for RFS in patients with NAT. A couple of previous studies demonstrated that reductions in the SUVmax were an accurate predictor of prognosis in patients with BRPC followed by NAT and surgery [29,30]. Not only the SUVmax, but also the SUVpeak, metabolic tumor volume (MTV) and total lesion glycolysis (TLG) are parameters that can be extracted from PET-CT. As the efficacy of the aforementioned parameters is being actively researched, it is expected that some of the values or combinations of them would be helpful in selecting appropriate candidates for conversion surgery in the near future.

The present study has several limitations. Above all, as the study was designed to be a multicenter retrospective study, the results are vulnerable to several types of bias. We aimed to focus on the outcomes of a specific surgical procedure, DP-CAR, but details on preoperative evaluation, the process of determining the surgical candidates, and the operative techniques were limited due to the study’s retrospective nature. Also, since a number of surgeons and physicians were involved in the management of the patients from each institution, the study cohort inevitably had high heterogeneity. However, the present study has some remarkable implications. This was the first research reporting outcomes of DP-CAR using a Korean nationwide database, which included a large number of patients. We described the details of the cohort and investigated factors related to surgical and oncologic outcomes in various aspects, particularly in consideration of NAT.

## 5. Conclusions

In conclusion, DP-CAR, despite its surgical risk and morbidity, is a procedure that many pancreatic surgeons will someday encounter since surgical resection still plays a major role in improving the survival of patients with pancreatic cancer. Our ongoing challenge will be to set the criteria for selecting optimal candidates of DP-CAR, to maximize the effect of multimodal treatment for pancreatic cancer.

## Figures and Tables

**Table 1:** Demographic and preoperative clinical data of the study cohort (*n* = 75).

Variables	N (%) or Mean (±SD)	Variables	N (%) or Mean (±SD)
Age at operation	62.6 (±8.8)	Initial resectability	
Sex		Resectable	2 (2.7)
Male	38 (50.6)	Borderline-resectable	42 (56.0)
Female	37 (49.4)	Locally-advanced	30 (40.0)
BMI (kg/m^2^)	23.6 (±2.9)	Unknown	1 (1.3)
ASA score			
I	7 (9.3)	*In NAT group (n = 42)*	
II	60 (80.0)	Initial resectability	
III	6 (8.0)	Borderline-resectable	18 (42.9)
Underlying cardiovascular disease	28 (37.3)	Locally-advanced	23 (54.8)
Underlying DM	33 (44.0)	Unknown	1 (2.3)
		Post-NAT resectability	
Preop. CA 19-9 (IU/mL)	164.7 (±419.2)	Borderline-resectable	26 (61.9)
Elevated (>37 IU/mL)	31 (41.3)	Locally-advanced	16 (38.1)
Preop. NLR	2.2 (±1.5)		
Preop. PLR	125.3 (±57.4)		
Neoadjuvant treatment	42 (56.0)		
Chemotherapy	27 (36.0)		
Chemo-radiotherapy	15 (20.0)		

N, number; SD, standard deviation; BMI, body mass index; ASA, the American Society of Anesthesiologists; DM, Diabetes Mellitus; Preop., preoperative; CA 19-9, carbohydrate antigen 19-9; NLR, neutrophil-to-lymphocyte ratio; PLR, platelet-to-lymphocyte ratio; NAT, neoadjuvant treatment.

**Table 2:** Operative findings and pathologic results of the patients (*n* = 75).

Variables	N (%) or Mean (±SD)	Variables	N (%) or Mean (±SD)
*Operative findings*		*Pathology*	
Arterial reconstruction		Tumor size, cm	3.6 (±1.6)
Aorta to CHA, with graft	2 (2.7)	LN metastasis	52 (69.3)
PV resection	20 (26.7)	Tumor differentiation	
		WD	7 (9.3)
Combined organ resection		MD	52 (69.3)
Colon	5 (6.7)	PD/Undifferentiated	12 (16.0)
Stomach	4 (5.3)	PNI	67 (89.3)
		LVI	35 (46.7)
Operation time, min	298.4 (±98.9)		
Estimated blood loss, mL	582.3 (±509.1)	AJCC 8th Stage	
RBC transfusion	18 (24.0)	No residual tumor	2 (2.7)
		IA/IB	7 (9.3)/8 (10.6)
		IIA/IIB	3 (4.0)/32 (42.7)
		III	18 (24.0)
		IV (M1 disease)	5 (6.7)
		R0 resection	49 (65.3)
		Pt. with upfront surgery	18 (54.5)
		Pt. with NAT	31 (73.8)
		Adjuvant treatment	54 (72.0)
		Chemotherapy	39 (52.0)
		Radiotherapy	2 (2.7)
		Chemo-radiotherapy	13 (173)

N, number; SD, standard deviation; CHA, common hepatic artery; PV, portal vein; RBC, red blood cell; LN, lymph node; WD, well differentiated; MD, moderately differentiated; PD, poorly differentiated; PNI, perineural invasion; LVI, lymphovascular invasion; AJCC, the American Joint Committee on Cancer; Pt., patients; NAT, neoadjuvant treatment.

**Table 3:** Postoperative complications and mortalities (*n* = 75).

Variables	N (%) or Mean (±SD)	Details on Mortality Cases
Overall complication	47 (62.7)	*Case #1—Procedure-related* F/81—Upfront surgery for BRPC CR-POPF and Gastric ischemia Sepsis—Multi organ failure Expired on POD #20
Complications, C-D grade ≥ 3	20 (26.7)	
Re-opeartion	2 (2.7)	
Overall 90-day mortality	3 (4.0)	
Procedure-related 90-day mortality	2 (2.7)	
	
Gastropathy	10 (13.3)	*Case #2—Procedure-related* M/70—Neoadjuvant CCRT for BRPC Septic shock due to CR-POPF Expired on POD #9
Delayed gastric emptying	6 (8.0)	
Gastric ischemia (requiring non-surgical management)	3 (4.0)	
Gastric perforation (requiring re-operation)	1 (1.3)	
Hepatic ischemia (requiring conservative management)	2 (2.7)	
Thrombotic occlusion—hepatic artery/reconstructed artery	0 (0)	
Thrombotic occlusion—PV	4 (5.3)	
		*Case #3* F/71—Neoadjuvant CCRT for LAPC Septic shock due to postop. pneumonia Expired on POD #20
CR-POPF	20 (26.7)	
POPF—grade B	17 (22.7)	
POPF—grade C	3 (4.0)	
	
Length of hospital stay, days	18.1 (±13.7)	
Re-admission within 90 days	14 (18.7)	

N, number; SD, standard deviation; C-D, Clavien-Dindo; PV, portal vein; CR-POPF, clinically relevant postoperative pancreatic fistula; BRPC, borderline resectable pancreatic cancer; POD, postoperative day; CCRT, concomitant chemoradiation therapy; LAPC, locally advanced pancreatic cancer.

**Table 4:** Univariate and multivariate analysis of risk factors for Clavien–Dindo grade ≥ 3 complications (*n* = 75).

Variables	Univariable *p*	OR	95% CI	Multivariable *p*
Age	0.129			
Sex, male (ref. female)	0.651			
BMI	0.928			
ASA score	0.161			
Underlying cardiovascular diseases	0.774			
Underlying Diabetes Mellitus	0.529			
Preop. elevated CA 19-9	0.152			
Preop. NLR	0.493			
Preop. PLR	0.958			
NAT—chemotherapy	0.031	4.445	1.176–16.795	0.028
NAT—CCRT	0.349	2.350	0.481–11.469	0.291
Portal vein resection	0.092	2.756	0.847–8.968	0.092
Arterial reconstruction	-			
Estimated blood loss	0.994			
RBC transfusion	0.103			

OR, odds ratio; CI, confidence interval; BMI, body mass index; ASA, the American Society of Anesthesiologists; Preop., preoperative; CA 19-9, carbohydrate antigen 19-9; NLR, neutrophil-to-lymphocyte ratio; PLR, platelet-to-lymphocyte ratio; NAT, neoadjuvant treatment; CCRT, concomitant chemoradiation therapy; RBC, red blood cell.

**Table 5:** Univariate and multivariate analysis of preoperative parameters for recurrence-free and overall survival, in patients with neoadjuvant treatment (*n* = 42).

Variables	Recurrence-Free Survival				Overall Survival			
	uni *p*	HR	95% CI	*p*	uni *p*	HR	95% CI	*p*
Age	0.765				0.151			
Sex, male (ref. female)	0.259				0.419			
BMI	0.526				0.587			
ASA score	0.118				0.615			
Underlying CVD	0.422				0.253			
Underlying DM	0.719				0.763			
Preop. elevated CA 19-9	0.065	3.047	0.987–9.409	0.053	0.185			
Preop. NLR	0.190				0.525			
Preop. PLR	0.908				0.524			
Preop. PV invasion *	0.719				0.702			
Preop. SUVmax (PET)	0.012	1.548	1.061–2.260	0.024	0.086	1.387	0.955–2.016	0.086
NAT regimen, FOLFIRINOX (ref. others)	0.817				0.115			
Combined RT	0.560				0.937			
Biologic response to NAT (decrease in CA 19-9)	0.456				0.021	0.114	0.017–0.772	0.026
Radiologic response to NAT (radiologic downstaging)	0.768				0.237			
Metabolic response to NAT (decrease in SUVmax)	0.473				0.251			

Uni *p*, *p*-value from univariable analysis; HR, hazard ratio; CI, confidence interval; BMI, body mass index; ASA, the American Society of Anesthesiologists; CVD, cardiovascular disease; DM, Diabetes Mellitus; Preop., preoperative; CA 19-9, carbohydrate antigen 19-9; NLR, neutrophil-to-lymphocyte ratio; PLR, platelet-to-lymphocyte ratio; PV, portal vein; SUVmax, maximal standardized uptake value; PET, positron emission tomography; NAT, neoadjuvant treatment; FOLFIRINOX, combination of fluorouracil, leucovorin, irinotecan, and oxaliplatin; RT, radiotherapy. * When a tumor was suspected of invading portal vein in preoperative images.

## Data Availability

The data presented in this study are available on request from the corresponding author. The data are not publicly available due to individual privacy.

## Supplementary Materials

The following supporting information can be downloaded at: https://www.mdpi.com/article/10.3390/cancers15153850/s1. Table S1. Previous reports on distal pancreatectomy with celiac axis resection.

## References

[1] Li D., Xie K., Wolff R., Abbruzzese J.L. (2004). Pancreatic cancer. Lancet.

[2] Van Veldhuisen E., van den Oord C., Brada L.J., Walma M.S., Vogel J.A., Wilmink J.W., Del Chiaro M., Van Lienden K.P., Meijerink M.R., Van Tienhoven G. (2019). Locally advanced pancreatic cancer: Work-up, staging, and local intervention strategies. Cancers.

[3] Michels N.A. (1953). Collateral arterial pathways to the liver after ligation of the hepatic artery and removal of the celiac axis. Cancer.

[4] Kondo S., Katoh H., Shimizu T., Omi M., Hirano S., Ambo Y., Okushiba S., Morikawa T. (2000). Preoperative embolization of the common hepatic artery in preparation for radical pancreatectomy for pancreas body cancer. Hepato-Gastroenterology.

[5] Hirai I., Kimura W., Kamiga M., Mizutani M., Takeshita A., Watanabe T., Fuse A. (2005). The significance of intraoperative Doppler ultrasonography in evaluating hepatic arterial flow when assessing the indications for the Appleby procedure for pancreatic body cancer. J. Hepato-Biliary-Pancreatic Surg..

[6] Yamamoto T., Satoi S., Kawai M., Motoi F., Sho M., Uemura K.-I., Matsumoto I., Honda G., Okada K.-I., Akahori T. (2018). Is distal pancreatectomy with en-bloc celiac axis resection effective for patients with locally advanced pancreatic ductal adenocarcinoma? -Multicenter surgical group study. Pancreatology.

[7] Klompmaker S., Peters N.A., Van Hilst J., Bassi C., Boggi U., Busch O.R., Niesen W., Van Gulik T.M., Javed A.A., the E-AHPBA DP-CAR study group (2019). Outcomes and Risk Score for Distal Pancreatectomy with Celiac Axis Resection (DP-CAR): An International Multicenter Analysis. Ann. Surg. Oncol..

[8] National Comprehensive Cancer Network NCCN Clinical Practice Guidelines in Oncology (NCCN Guidelines^®^) for Pancre-atic Adenocarcinoma V.1.2020. https://www.nccn.org/guidelines/nccn-guidelines.

[9] Bassi C., Marchegiani G., Dervenis C., Sarr M., Hilal M.A., Adham M., Allen P., Andersson R., Asbun H.J., Besselink M.G. (2017). The 2016 update of the International Study Group (ISGPS) definition and grading of postoperative pancreatic fistula: 11 Years After. Surgery.

[10] Appleby L.H. (1953). The coeliac axis in the expansion of the operation for gastric carcinoma. Cancer.

[11] Hishinuma S., Ogata Y., Tomikawa M., Ozawa I. (2007). Stomach-Preserving Distal Pancreatectomy with Combined Resection of the Celiac Artery: Radical Procedure for Locally Advanced Cancer of the Pancreatic Body. J. Gastrointest. Surg..

[12] Schmocker R.K., Wright M.J., Ding D., Beckman M.J., Javed A.A., Cameron J.L., Lafaro K.J., Burns W.R., Weiss M.J., He J. (2020). An Aggressive Approach to Locally Confined Pancreatic Cancer: Defining Surgical and Oncologic Outcomes Unique to Pancreatectomy with Celiac Axis Resection (DP-CAR). Ann. Surg. Oncol..

[13] Nakamura T., Hirano S., Noji T., Asano T., Okamura K., Tsuchikawa T., Murakami S., Kurashima Y., Ebihara Y., Nakanishi Y. (2016). Distal Pancreatectomy with en Bloc Celiac Axis Resection (Modified Appleby Procedure) for Locally Advanced Pancreatic Body Cancer: A Single-Center Review of 80 Consecutive Patients. Ann. Surg. Oncol..

[14] Baumgartner J.M., Krasinskas A., Daouadi M., Zureikat A., Marsh W., Lee K., Bartlett D., Moser A.J., Zeh H.J. (2012). Distal Pancreatectomy with En Bloc Celiac Axis Resection for Locally Advanced Pancreatic Adenocarcinoma Following Neoadjuvant Therapy. J. Gastrointest. Surg..

[15] Gong H., Ma R., Gong J., Cai C., Song Z., Xu B. (2016). Distal Pancreatectomy With En Bloc Celiac Axis Resection for Locally Advanced Pancreatic Cancer. Medicine.

[16] Yoshiya S., Fukuzawa K., Inokuchi S., Kosai-Fujimoto Y., Sanefuji K., Iwaki K., Motohiro A., Itoh S., Harada N., Ikegami T. (2019). Efficacy of Neoadjuvant Chemotherapy in Distal Pancreatectomy with En Bloc Celiac Axis Resection (DP-CAR) for Locally Advanced Pancreatic Cancer. J. Gastrointest. Surg..

[17] Yoshitomi H., Sakai N., Kagawa S., Takano S., Ueda A., Kato A., Furukawa K., Takayashiki T., Kuboki S., Miyzaki M. (2019). Feasibility and safety of distal pancreatectomy with en bloc celiac axis resection (DP-CAR) combined with neoadjuvant therapy for borderline resectable and unresectable pancreatic body/tail cancer. Langenbeck’s Arch. Surg..

[18] Murakami Y., Nakagawa N., Kondo N., Hashimoto Y., Okada K., Seo S., Otsuka H. (2021). Survival impact of distal pancreatectomy with en bloc celiac axis resection combined with neoadjuvant chemotherapy for borderline resectable or locally advanced pancreatic body carcinoma. Pancreatology.

[19] Egorov V., Kim P., Kharazov A., Dzigasov S., Popov P., Rykova S., Zelter P., Demidova A., Kondratiev E., Grigorievsky M. (2022). Hemodynamic, Surgical and Oncological Outcomes of 40 Distal Pancreatectomies with Celiac and Left Gastric Arteries Resection (DP CAR) without Arterial Reconstructions and Preoperative Embolization. Cancers.

[20] Denecke T., Andreou A., Podrabsky P., Grieser C., Warnick P., Bahra M., Klein F., Hamm B., Neuhaus P., Glanemann M. (2010). Distal Pancreatectomy With En Bloc Resection of the Celiac Trunk for Extended Pancreatic Tumor Disease: An Interdisciplinary Approach. Cardiovasc. Interv. Radiol..

[21] Ueda A., Sakai N., Yoshitomi H., Furukawa K., Takayashiki T., Kuboki S., Takano S., Suzuki D., Kagawa S., Mishima T. (2019). Is hepatic artery coil embolization useful in distal pancreatectomy with en bloc celiac axis resection for locally advanced pancreatic cancer?. World J. Surg. Oncol..

[22] Abo D., Hasegawa Y., Sakuhara Y., Terae S., Shimizu T., Tha K.K., Tanaka E., Hirano S., Kondo S., Shirato H. (2011). Feasibility of a dual microcatheter-dual interlocking detachable coil technique in preoperative embolization in preparation for distal pancreatectomy with en bloc celiac axis resection for locally advanced pancreatic body cancer. J. Hepato-Biliary-Pancreatic Sci..

[23] Springfeld C., Ferrone C.R., Katz M.H.G., Philip P.A., Hong T.S., Hackert T., Büchler M.W., Neoptolemos J. (2023). Neoadjuvant therapy for pancreatic cancer. Nat. Rev. Clin. Oncol..

[24] van Veldhuisen E., Vogel J.A., Klompmaker S., Busch O.R., van Laarhoven H.W., van Lienden K.P., Wilmink J.W., Marsman H.A., Besselink M.G. (2018). Added value of CA19-9 response in predicting resectability of locally advanced pancreatic cancer following induction chemotherapy. HPB.

[25] Heger U., Sun H., Hinz U., Klaiber U., Tanaka M., Liu B., Sachsenmaier M., Springfeld C., Michalski C.W., Büchler M.W. (2020). Induction chemotherapy in pancreatic cancer: CA 19-9 may predict resectability and survival. HPB.

[26] Schwartz L.H., Litière S., de Vries E., Ford R., Gwyther S., Mandrekar S., Shankar L., Bogaerts J., Chen A., Dancey J. (2016). RECIST 1.1—Update and clarification: From the RECIST committee. Eur. J. Cancer.

[27] Katz M.H.G., Fleming J.B., Bhosale P., Varadhachary G., Lee J.E., Wolff R., Wang H., Abbruzzese J., Pisters P.W.T., Vauthey J.-N. (2012). Response of borderline resectable pancreatic cancer to neoadjuvant therapy is not reflected by radiographic indicators. Cancer.

[28] Barreto S.G., Loveday B., Windsor J.A., Pandanaboyana S. (2017). Detecting tumour response and predicting resectability after neoadjuvant therapy for borderline resectable and locally advanced pancreatic cancer. ANZ J. Surg..

[29] Akita H., Takahashi H., Ohigashi H., Tomokuni A., Kobayashi S., Sugimura K., Miyoshi N., Moon J.-H., Yasui M., Omori T. (2017). FDG-PET predicts treatment efficacy and surgical outcome of pre-operative chemoradiation therapy for resectable and borderline resectable pancreatic cancer. Eur. J. Surg. Oncol. (EJSO).

[30] Lee W., Oh M., Kim J.S., Park Y., Kwon J.W., Jun E., Song K.B., Lee J.H., Hwang D.W., Yoo C. (2021). Metabolic activity by FDG-PET/CT after neoadjuvant chemotherapy in borderline resectable and locally advanced pancreatic cancer and association with survival. Br. J. Surg..

