# Supplementary material for: 15-Year Experience of Distal Pancreatectomy with Celiac Axis Resection (DP-CAR) for Pancreatic Cancer—A Korean Nationwide Investigation
Source: Cancers (Basel). 2023 Jul 28;15(15):3850. doi: 10.3390/cancers15153850 (PMC10417433; doi:10.3390/cancers15153850)
Supplement: Supplementary file 1 [file cancers-15-03850-s001.zip › cancers-2490509-supplementary.pdf]

Table S1. Previous reports on distal pancreatectomy with celiac axis resection.

Study group (year)	N	R0 rate	CD ≥ 3 complications (Mortality)	RFS (median)	OS (median)	Remarks
Japan[6] (2018)	72	67.0 %	41.7 % (4.2 %)	9.3 m	17.5 m	NAT: 56.0 %
E-AHPBA[7] (2019)	71	55.0 %	41.0 % (16.0 %)	n/a	19.0 m	NAT: 51.0 %
The U.S.[12] (2021)	54	87.0 %	43.0 % (2.0 %)	9.0 m	25.0 m	NAT: 98.2 %
Russia[11] (2022)	40	91.2 %	42.5 % (7.5 %)	19.0 m	29.0 m	NAT: 53.0 % Including 34 patients with pancreatic cancer (others: gastric cancer, neuroendocrine tumor, and lymphoma)
Korea (2023)	75	65.3 %	26.7 % (2.7 %)	7.0 m	19.0 m	NAT: 56.0 %

N, the number of included patients; CD, Clavien–Dindo grade; RFS, recurrence-free survival; OS, overall survival; m, months; NAT, neoadjuvant treatment
